# Chemotherapy-associated hemorrhagic posterior reversible encephalopathy syndrome (PRES) with considerations for circle of Willis variants on cerebral blood flow and autoregulation: A case report

**DOI:** 10.1097/MD.0000000000037250

**Published:** 2024-02-23

**Authors:** Bahadar S. Srichawla, Kendall Presti, Vincent Kipkorir, Idanis Berrios Morales

**Affiliations:** a Department of Neurology, University of Massachusetts Chan Medical School, Worcester, MA; b Department of Medicine, University of Nairobi, Nairobi, Kenya.

**Keywords:** cancer, chemotherapy, circle of Willis variants, fetal-type posterior cerebral artery, hemorrhage, Hodgkin lymphoma, posterior reversible encephalopathy syndrome, PRES

## Abstract

**Rationale:**

Hodgkin lymphoma, a lymphatic system cancer, is treated by chemotherapy, radiation therapy, and hematopoietic stem cell transplantation. Posterior reversible encephalopathy syndrome (PRES) is a rare neurotoxic effect associated with several drugs and systemic conditions. This case study emphasizes the potential risks of intensive chemotherapy regimens and postulates the impact of the circle of Willis variants on the heterogeneity of hemispheric lesions in PRES.

**Patient concerns:**

A 42-year-old woman diagnosed with stage IIA nodular sclerosing Hodgkin lymphoma and chronic thrombocytopenia presented after 6 years of initial diagnosis and 4 years post-haploidentical transplant. She underwent planned chemotherapy with ifosfamide, carboplatin, and etoposide.

**Diagnoses:**

She developed an alteration in her mental status. A computerized tomography scan and angiogram of the head and neck revealed findings consistent with PRES and a left fetal-type posterior cerebral artery with an aplastic A_1_ segment of the left anterior cerebral artery. One hour later she was found comatose with clinical sequelae of an uncal herniation.

**Interventions:**

Subsequent events led to emergent intubation, and administration of 23.4% hypertonic saline. A repeat computerized tomography scan showed a right intraparenchymal hemorrhage with fluid-fluid levels measuring up to 4.7 cm, bilateral subarachnoid hemorrhage, right uncal herniation, and 15 mm of leftward midline shift. She emergently underwent a right decompressive hemi-craniectomy.

**Outcomes:**

An magnetic resonance imaging of the brain demonstrated bilateral cytotoxic edema involving the parieto-occipital lobes. Despite interventions, the patient’s neurological condition deteriorated, leading to a declaration of brain death on the 8th day.

**Lessons:**

This case underscores the importance of recognizing the severe neurological complications, including PRES, associated with chemotherapeutic treatments in Hodgkin lymphoma. PRES may also be exacerbated by coagulopathies such as thrombocytopenia in this case. The circle of Willis variants may influence cerebral blood flow, autoregulation, and other factors of hemodynamics, leading to increased susceptibility to both radiographic lesion burden and the worst clinical outcomes.

## 1. Introduction

Hodgkin lymphoma is a type of lymphoma that originates in the lymphatic system.^[1]^ Treatment modalities include chemotherapy, radiation therapy, and in refractory cases, hematopoietic stem cell transplantation. Although these treatments have shown significant efficacy in inducing remission and prolonging survival, they are not without complications.^[2]^ Neurotoxicity is one of the rare but serious side effects of some chemotherapeutic agents, with presentations ranging from subtle cognitive changes to severe life-threatening conditions.^[3]^ Posterior reversible encephalopathy syndrome (PRES) is a neurologic condition characterized by a myriad of symptoms, ranging from headache and visual disturbances to seizures and altered consciousness. PRES has been associated with various medications and systemic conditions, including chemotherapy.^[4]^ The pathophysiology of this syndrome is not yet understood, although hypertension and endothelial dysfunction play a significant role.^[5]^ We depict the unfortunate case of a 42-year-old woman with Hodgkin lymphoma, who after intensive chemotherapy, developed PRES leading to catastrophic neurological events, offering insight into the potential hazards of aggressive chemotherapeutic regimens. Furthermore, we hypothesize the influence of the circle of Willis variants and hemodynamic changes that may influence the heterogeneity and severity of the burden of hemispheric lesion in PRES. This case report was completed according to the CARE (CAse REport) Statement and Checklist.^[6]^

## 2. Case presentation

A 42-year-old woman with a medical history of stage IIA nodular sclerosing Hodgkin lymphoma presented to the hospital for ifosfamide, carboplatin, and etoposide (ICE) therapy. She was diagnosed with Hodgkin lymphoma 6 years ago and had a haploidentical transplant 4 years ago. She completed 6 cycles of doxorubicin, bleomycin, vinblastine, and dacarbazine chemotherapy. Her malignancy subsequently relapsed and had completed ICE therapy. She continued to remain in remission for 4 years, however a positron emission tomography/computerized tomography (CT) scan prior to her current admission showed high-disease burden above and below the diaphragm and in extra-nodal locations. An excisional lymph node biopsy confirmed recurrence of Hodgkin lymphoma. Initial laboratory tests including a complete blood count, and comprehensive metabolic panel demonstrated a sodium of 127 mmol/L, chloride 89 mmol/L, white blood cell count 1.4 × 10^3^/μL, hemoglobin 9.2 g/dL, platelets 24 × 10^3^/μL, and uric acid of 2.0 mg/dL.

**Figure 1.**
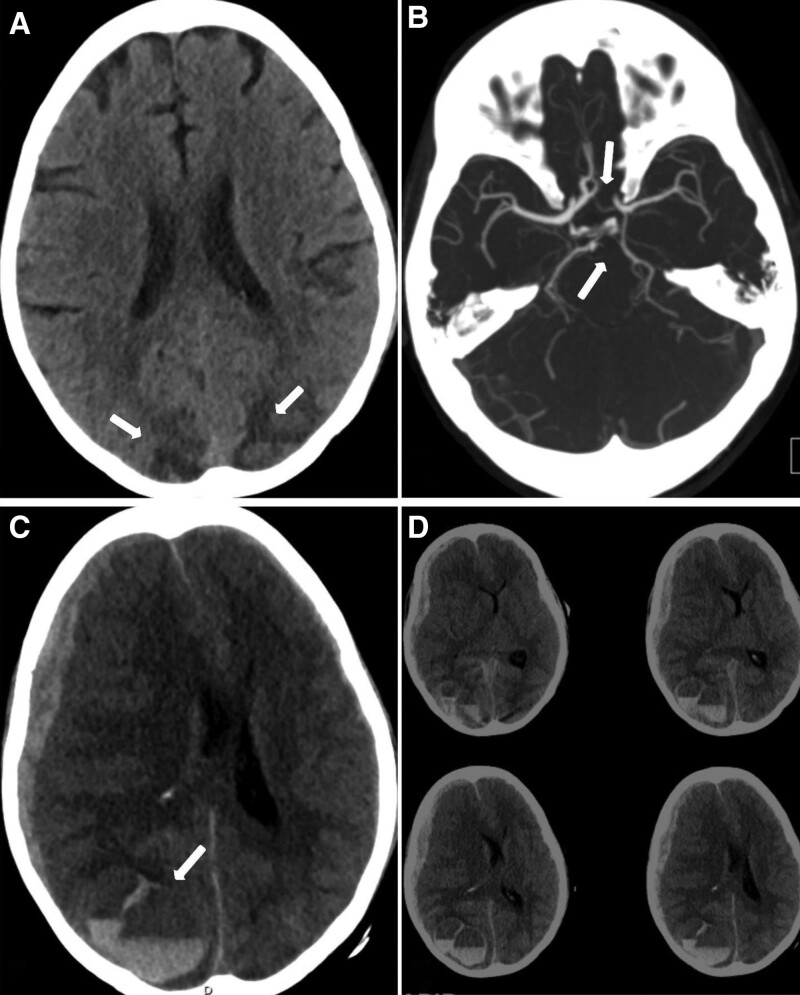
(A) Initial CT scan of the head demonstrating bilateral parieto-occipital hypodensities with mixed hyperdensity signal concerning for PRES. (B) CT angiogram axial view of the circle of Willis. Top arrow depicting an aplastic A_1_ segment of the left anterior cerebral artery (ACA). Bottom arrow showing a fetal origin of the left posterior cerebral artery (fPCA). (C) Repeat CT scan axial view of the head 1-hour after the initial scan showing significant fluid-fluid levels (arrow) representative of thrombocytopenia related intraparenchymal hemorrhage in PRES. Additionally, a 15 mm leftward midline shift, bilateral subdural hematomas, and right uncal herniation. (D) Multi-slice images detecting intracerebral hemorrhage. CT = computerized tomography, PRES = posterior reversible encephalopathy syndrome.

**Figure 2.**
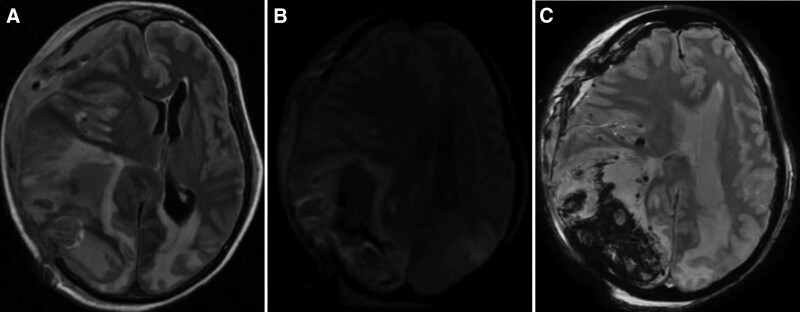
(A) MRI of the brain T2-FLAIR sequence demonstrating bilateral parieto-occipital hyperintensities significant for cytotoxic edema consistent with PRES. (B) DWI sequence with hyperintensities depicting areas of acute ischemia. ADC correlation present. (C) SWI sequence showing areas of hypointensity within the right cerebral hemisphere representative of the intraparenchymal hemorrhage. ADC = apparent diffusion coefficient, DWI = diffusion weighted imaging, MRI = magnetic resonance imaging, PRES = posterior reversible encephalopathy syndrome, SWI = susceptibility weighted imaging.

She completed the ICE chemotherapy regimen on day 1 of her presentation. However, on day 2 a code stroke was activated for altered mental status. A CT scan of the head revealed bilateral hypodensities with mixed hyperdensities in the bilateral parieto-occipital lobes concerning for PRES. A CT angiogram of the head and neck showed a left fetal posterior cerebral artery (fPCA) and aplastic A_1_ segment of the left anterior cerebral artery (ACA) (Fig. 1A and B). One-hour later a repeat code stroke was activated, and the neurological exam showed a fixed dilated blown right 6 mm pupil and pin-point 1 mm left pupil both unreactive to light. Her Glasgow coma scale was <9 and she was emergently intubated and was given 1 dose of 30 mL of 23.4% hypertonic saline. A repeat CT scan of the head showed a right intraparenchymal hemorrhage with fluid-fluid levels measuring up to 4.7 cm, bilateral subarachnoid hemorrhage, right uncal and subfalcine herniations, and bilateral subdural hematoma and 15 mm of leftward midline shift (Fig. 1C and D). The patient was transferred to the neurological intensive care unit and was taken for an emergent right decompressive hemi-craniectomy. Unfortunately, her midline shift did not improve, and her hemorrhage had progressed. She continued on frequent serial neurological examinations and osmolar therapy with hypertonic saline and mannitol. On physical examination her Full Outline of UnResponsiveness score was 4 (E0 M2 B2 R0).^[7]^ A magnetic resonance imaging study of her brain showed cytotoxic edema in left temporal and superior frontal regions as well as bilateral parieto-occipital regions consistent with PRES (Fig. 2). Serial CT scans were completed and demonstrated loss of gray-white matter differentiation and diffuse cerebral edema. An electroencephalogram was completed revealing global continuous suppression <10 μV, consistent with severe generalized cerebral dysfunction. Her neurological exam deteriorated over the course of 1-week to a Full Outline of UnResponsiveness score of zero (E0 M0 B0 R0) and on the 8th day of her presentation 2 brain death examinations were completed and the patient was terminally extubated.

## 3. Discussion

Hodgkin lymphoma, while responding to a wide variety of treatments, is not without the risks associated with its therapeutic regimens.^[1]^ Neurotoxicity, as seen in this case, represents a severe and potentially fatal complication. The development of PRES after chemotherapy, especially after an intensive regimen such as ICE, emphasizes the need to approach therapeutic strategies with vigilance, bearing in mind the balance between therapeutic efficacy and potential risks. It is worth noting that the catastrophic neurological deterioration of the patient, while initially consistent with PRES, rapidly evolved to include severe intraparenchymal hemorrhage, herniations, and cerebral edema leading to brain death. This severe progression is rarely seen with PRES, suggesting a possible multifactorial etiology, including the impact of vascular abnormalities and the effects of chemotherapy on immune-mediated cerebrovascular endothelial injury. The presence of intraparenchymal hemorrhage and subdural hematoma is believed to be significantly influenced by the patient’s chronic thrombocytopenia. To support this hypothesis the second CT scan of the head demonstrated fluid-fluid levels.^[8]^ The presence of fluid-fluid levels indicates an ongoing coagulopathy such as thrombocytopenia or anticoagulation use and represents the layering of blood within the site of injury.^[9]^

The pathophysiology of chemotherapy-associated PRES is still a matter of debate. However, current proposed mechanisms include direct neurotoxicity of endothelial cells lining the arterioles, which play a critical role in blood-brain barrier integrity and cerebral autoregulation.^[10]^ The resulting injury can lead to vasogenic edema and ischemia. In addition, cisplatin (platinum-based) and bleomycin have been shown to exhibit laxity of the blood-brain barrier.^[11,12]^ A previous systematic review demonstrated that women are more likely to be diagnosed with chemotherapy-associated PRES (77%), with seizures and altered mental status being the most common presenting symptom. And 93% of the cases demonstrated radiographic lesions within the posterior circulation. Most of the cases (74%) were individuals on the rituximab, cyclophosphamide, doxorubicin, vincristine, and prednisolone chemotherapy regimen (rituximab, cyclophosphamide, doxorubicin, vincristine, and prednisolone).^[4]^ Gemcitabine and platinum-based drugs, such as cisplatin, are also commonly encountered agents causing PRES.^[11,12]^ In our case the patient was on carboplatin, a platinum-based agent. The management of PRES involves immediate cessation of the offending agent and strict blood pressure control. In addition, some have used steroids to reduce inflammation and edema in PRES. However, high-dose steroids themselves have been reported to cause PRES, therefore caution is recommended for the use of steroids for treatment.^[13]^ Hemorrhagic conversion is an established and devastating consequence of PRES.^[14]^ The pathophysiology is not fully understood, but the proposed mechanisms include (1) reperfusion injury as cerebral blood flow (CBF) increases after initial ischemic injury, (2) progression of cerebral dysautoregulation causing cerebral vasospasm and extravasation of red blood cells, as well as (3) direct neurotoxicity of cerebral blood vessels leading to rupture and subsequent intracerebral hemorrhage.^[14]^ A previous study of 151 patients with PRES demonstrated a 12.5% hemorrhagic conversion rate. And immunosuppression was the most frequently observed comorbidity (22%).^[15]^ Hemorrhagic PRES can range from focal hemorrhage, convexal subarachnoid hemorrhage to micro-hemorrhages seen on susceptibility weighted imaging.^[15,16]^ In our case thrombocytopenia, history of allo-bone marrow transplantation served as an exacerbating factor leading to severe intracranial hemorrhage in correlation with prior studies on hemorrhagic PRES risk factors.^[15,16]^ However, to understand why this hemorrhage was unilaterally severe when the initial lesions associated with PRES were symmetric on the initial head CT scan requires a more in-depth understanding of cerebral hemodynamics and the influence of anatomical variants.

PRES, classically, is marked by its reversible nature, and while the exact etiology remains elusive, it is generally agreed that the syndrome is related to impaired cerebral autoregulation and endothelial dysfunction.^[5]^ Cerebral autoregulation is a crucial physiological mechanism that maintains relatively constant CBF over a range of systemic arterial blood pressures.^[17]^ It is achieved through dynamic constriction or dilation of the cerebral arterioles in response to changes in perfusion pressure, ensuring stable delivery of oxygen and nutrients to the brain. Dysfunction in this autoregulation can lead to overperfusion or hypoperfusion, both of which can damage the delicate vascular structures of the brain and the blood-brain barrier.^[18]^ PRES is believed to be associated with this dysfunction. In the context of PRES, acute elevations in blood pressure are hypothesized to exceed the upper limit of autoregulation, leading to hyperperfusion. This hyperperfusion, in turn, can result in endothelial injury, disruption of the blood-brain barrier, and consequent vasogenic edema, especially in the posterior cerebral regions that are less protected by sympathetic innervation.^[19]^ However, there are also cases of PRES where hypoperfusion due to vasoconstriction is suggested as the primary mechanism. Impaired or reduced CBF has been observed in some cases of PRES. Reduced perfusion in PRES suggests that vasoconstriction, rather than vasodilation, might be a primary mechanism in certain patients. This hypoperfusion can result from intense cerebral arteriolar constriction, leading to ischemia and subsequent vasogenic edema.^[20]^ Other systemic conditions associated with this hypoperfusion injury in the brain include pancreatitis-mediated infarction of the lateral geniculate bodies.^[21]^ The involvement of endothelial dysfunction and the release of vasoconstrictive substances might contribute to this vasoconstriction. Hypomagnesemia has been identified as an independent and/or exacerbating factor that leads to PRES due to the critical role of magnesium in maintaining endothelial function.^[12,22]^ The exact pathophysiological underpinnings remain a topic of ongoing investigation, but the consensus is that altered cerebral autoregulation plays a central role in the development of this syndrome.

The circle or polygon of Willis, a vascular structure at the base of the brain, is instrumental in distributing arterial blood to the cerebrum. The fPCA and the aplastic A_1_ segment of the ACA refer to variations in the development of the cerebral arteries. The fPCA configuration occurs when the posterior cerebral artery receives its blood supply from the anterior circulation rather than the basilar artery, indicating the persistence of embryonic connections. The aplastic A_1_ is when the initial segment of the anterior cerebral artery is absent or hypoplastic, which may lead to altered hemodynamic flow within the anterior circulation of the brain. Both conditions are generally asymptomatic and often incidental, but they can be of clinical significance in the context of cerebral ischemia or during neurosurgical procedures. The fPCA has already been implicated in the influence of cerebral ischemia of the posterior circulation.^[23]^ Furthermore, a partial and full fPCA has been associated with 1.448 and 3.027 odds of having an ischemic stroke, respectively.^[24]^ These circle of Willis anomalies might influence cerebrovascular resistance (CVR) in the brain regions they supply. Mathematically, CBF can be described by the equation CBF = CPP/CVR, where CPP stands for cerebral perfusion pressure. Thus, CVR is inversely related to CBF; a decrease in CVR would lead to an increase in CBF, provided that CPP remains constant.^[25]^ Emmert et al studied 385 individuals and determined that approximately 24% of them had a fetal-type variant posterior cerebral artery (PCA). Using CO_2_ challenge to derive relative CBF and cerebrovascular reactivity maps, it was determined that a fPCA was associated with an ipsilateral decrease in reactivity compared to the contralateral hemisphere. This decrease was most prominent in the temporal lobe. In addition, CBF was decreased in the bilateral watershed areas.^[26]^ Noorbakhsh et al performed a review of arterial spin labeling imaging in patients with fPCA. Eighty patients were found and 13/80 (16.2%) had hypoperfusion in the PCA territory contralateral to the side of the fPCA.^[27]^ Given this finding, the presence of a fPCA and/or an aplastic A_1_ segment of the ACA could potentially create a maladaptive CBF profile, and dysautoregulation leading to PRES. In the setting of PRES, where endothelial injury and disruption of the blood-brain barrier are paramount, regions with improved CBF could be less susceptible to drastic hemodynamic shifts and subsequent hemorrhagic transformations. This is of particular importance as PRES most commonly affects the parieto-occipital and temporal lobe junctions, which are commonly innervated by the posterior cerebral artery. However, more studies are needed to assess the effects of the circle of Willis variants on cerebral blood flow in both the ipsilateral and contralateral hemispheres and their potential role in disease states of dysautoregulation, including reversible cerebral vasoconstriction syndrome and PRES.

The primary limitation of this case study is its inherent nature: based on a single patient’s experience, it lacks generalizability to the broader population. Consequently, while offering valuable information, the findings may not be applicable to all patients with similar conditions or undergoing similar treatments. Additionally, as with most case studies, causation cannot be definitively established; only associations can be observed. The absence of a control or comparison group further prevents us from drawing robust conclusions. Last, there may have been unrecorded or unobserved confounding variables that influenced the patient’s clinical course, making it challenging to attribute observed results solely to the described factors.

## 4. Conclusions

This case underscores the complex interaction between aggressive chemotherapeutic regimens, the resultant hemorrhagic PRES, and the influence of vascular anatomical variations on cerebral hemodynamics. Additionally, allo-bone marrow transplantation, and thrombocytopenia may increase the risk of hemorrhagic PRES. The presence of a fetal-type PCA and an aplastic A_1_ ACA may alter cerebral blood flow potentially influencing both susceptibility and radiographic as well as clinical burden of PRES. Recognizing the potential hazards of intensive chemotherapy regimens and understanding the cerebral vascular anatomy of the individual patient could be crucial to predicting and managing neurotoxic complications effectively and should be a focus of future research in PRES.

## Author contributions

**Conceptualization:** Bahadar S. Srichawla, Kendall Presti, Vincent Kipkorir, Idanis Berrios Morales.

**Data curation:** Bahadar S. Srichawla.

**Formal analysis:** Bahadar S. Srichawla.

**Funding acquisition:** Bahadar S. Srichawla.

**Investigation:** Bahadar S. Srichawla, Idanis Berrios Morales.

**Methodology:** Bahadar S. Srichawla.

**Project administration:** Bahadar S. Srichawla.

**Resources:** Bahadar S. Srichawla.

**Software:** Bahadar S. Srichawla.

**Supervision:** Bahadar S. Srichawla, Idanis Berrios Morales.

**Validation:** Bahadar S. Srichawla.

**Visualization:** Bahadar S. Srichawla.

**Writing – original draft:** Bahadar S. Srichawla, Kendall Presti, Vincent Kipkorir, Idanis Berrios Morales.

**Writing – review & editing:** Bahadar S. Srichawla, Kendall Presti, Vincent Kipkorir, Idanis Berrios Morales.
